# Dental age estimation in Somali children and sub-adults combining permanent teeth and third molar development

**DOI:** 10.1007/s00414-019-02053-w

**Published:** 2019-04-16

**Authors:** Mari Metsäniitty, Janna Waltimo-Sirén, Helena Ranta, Steffen Fieuws, Patrick Thevissen

**Affiliations:** 1 Department of Forensic Medicine, University of Helsinki and National Institute for Health and Welfare, Kytösuontie 11, 00300 Helsinki, Finland; 2 Department of Oral and Maxillofacial Diseases, University of Helsinki and Helsinki University Hospital, Kytösuontie 9, 00014 Helsinki, Finland; 3 Department of Forensic Medicine, University of Helsinki, Kytösuontie 11, 00300 Helsinki, Finland; 4 Leuven Biostatistics and Statistical Bioinformatics Centre (L-BioStat), University of Leuven, Kapucijnenvoer 35, 3000 Leuven, Belgium; 5 Department of Imaging & Pathology, Forensic Odontology, KU Leuven Campus Saint Raphael 7, Block A, box 7001, 3000 Leuven, Belgium

**Keywords:** Forensic odontology, Forensic age estimation, Age determination, Dental development, Somali, Bayesian age estimation

## Abstract

Estimation of an individual’s age has important applications in forensics. In young individuals, it often relies on separate evaluations of permanent teeth (PT) and third molars (TM) development. Here, we analysed the age prediction performance of combined information from PT and TM in an unusual sample of healthy Somalis, born and living in Finland. PT development was staged according to Demirjian et al. (*Hum Biol*, 1973) and TM development according to Köhler et al. (*Ann Anat*, 1994), using panoramic radiographs from 803 subjects (397 males, 406 females) aged 3–23 years. A sex-specific Bayesian age-estimation model for the multivariate distribution of the stages conditional on age was fitted on PT, TM and PT and TM combined. The age-estimation performances were validated and quantified. The approach combining PT and TM only overestimated age with an ME of − 0.031 years in males and − 0.011 years in females, indicating the best age prediction performance.

## Introduction

Forensic age assessment among the living is mainly needed in cases where the real age of unaccompanied asylum seekers is unknown, or in criminal proceedings [1] and sometimes competitive sports [2]. In 2017, 68.5 million people had to forcibly leave their homes due to wars, violence and persecution [3] and in EU countries, of all minor asylum applicants 13% were unaccompanied [4]. In the future, a continuous need for forensic age estimation of unaccompanied minors with doubted age documentation is expected worldwide [5]. In forensic age assessment, dental development evaluation is a principal and widely used tool [6, 7]. Previous studies have demonstrated significant correlations between consecutive stages of dental development and chronological age [8–21].

Improved age-estimation results are obtained by combining diverse age predictors––frequently, dental and skeletal parameters [1, 22–26]. Moreover, in children and sub-adults, permanent teeth (PT) and third molars (TM) development have been used as combined predictors [27–30]. Analyses of models for PT, TM, and combined PT + TM have been tested on United Arab Emirati [27], Brazilian [28], Japanese [29], and Malaysian children [30], but to our knowledge not in populations of Black African origin.

The wide prediction intervals in age estimations based on third molar development are problematic (mean width of 95% prediction intervals approximately 6 years) [9, 31, 32]. Recent scientific evidence shows that ethnic differences are minimal in age estimations based on tooth development [11, 33, 34]. Therefore, when using third molars for age estimation, in absence of population-specific data, reference data with an age range covering the whole developmental track and a uniform age distribution are recommended rather than a population-specific reference sample [8, 11, 32, 35]. A benefit of the Bayesian approach is that it does not show a tendency of attraction to the middle (overestimating the young, underestimating the old), as is the case in regression models [9, 36–39].

In the literature, controversies still exist regarding possible differences in the dental development between Black and other populations [12, 40–42], although more and more evidence of equality has been appearing [11, 20, 33, 35, 43–46]. Studies on the dental development in sub-Saharan populations and in Somalis, indigenous to Northeast Africa, are few, however [12, 41, 47]. Recently, permanent tooth development has been studied in the South-African Black population [46], in Botswanan children [40], and in Somali children born in Finland [44]. Studies of third molar development in sub-Saharan populations were also recently published on South African [35, 43, 48–50], Sudanese [20], Kenyan [45], Botswanan [51], Nigerian and Senegalese individuals [35], and on Northeast Africans from Libya [52], Morocco [53] and Sudan [20].

The study aim was to analyse the age prediction performance of permanent teeth and third molar development, separately and combined, using a Bayesian approach, to detect if and in what age-groups added tooth information improves the accuracy of age prediction. These were analysed using a unique material—Somali children and subadults, born and living in Finland, with reliable data on chronological age and general health.

## Materials and methods

The Research Ethics Committee of the Hjelt Institute, University of Helsinki, Finland granted ethical approval (no. 02/2010), and the division of Oral Health Care of the Department of Social Services and Health Care in Helsinki, Finland provided the research permit (#HEL 2015–010918). All individuals included were born in Finland after 1.1.1980, had permanent residence in Helsinki, spoke Somali as their native language and both of their parents were born in Somalia. A search for individuals fitting the criteria was performed by the Finnish Population Register Centre, and 2115 individuals were discovered. From 811 of those individuals, 1231dental panoramic radiographs taken for dental care purposes were found in the division of Oral Health Care at the Department of Social Services and Health Care in Helsinki. Medical abnormalities, possibly affecting dental development, were an exclusion criterion. Only one dental panoramic radiograph per eligible individual was selected, aiming at a homogenous age distribution. The final studied material included dental panoramic radiographs from 803 subjects (397 males, 406 females) in the age range from 3 to 23 years (Table 1).

**Table 1:** Age and sex distribution of the Somali sample

Age (years)	Males	Percent	Females	Percent	Total	Percent
3–3.99	1	0.25	0	0.00	1	0.12
4–4.99	3	0.76	1	0.25	4	0.50
5–5.99	8	2.02	6	1.48	14	1.74
6–6.99	19	4.79	20	4.93	39	4.86
7–7.99	37	9.32	47	11.58	84	10.46
8–8.99	48	12.09	50	12.32	98	12.20
9–9.99	51	12.85	50	12.32	101	12.58
10–10.99	49	12.34	36	8.87	85	10.59
11–11.99	43	10.83	36	8.87	79	9.84
12–12.99	38	9.57	27	6.65	65	8.09
13–13.99	27	6.80	31	7.64	58	7.22
14–14.99	17	4.28	20	4.93	37	4.61
15–15.99	17	4.28	22	5.42	39	4.86
16–16.99	17	4.28	17	4.19	34	4.23
17–17.99	8	2.02	11	2.71	19	2.37
18–18.99	7	1.76	14	3.45	21	2.62
19–19.99	0	0.00	8	1.97	8	1.00
20–20.99	3	0.76	7	1.72	10	1.25
21–21.99	2	0.50	3	0.74	5	0.62
22–22.99	1	0.25	0	0.00	1	0.12
23–23.99	1	0.25	0	0.00	1	0.12
Total	397	100	406	100	803	100

The development of the seven left mandibular permanent teeth (PT; World Dental Federation (FDI) 31 to 37) was staged according to Demirjian et al. [19]. The technique is universally applied to permanent teeth [34, 54]. When one or more index teeth were missing, the contra-lateral homologous teeth were staged. The development of all present third molars (TM) were staged according to the 10-point staging technique developed by Gleiser and Hunt [55] and modified by Köhler et al. [21]. Köhler staging has been shown to be most suitable for age predictions in the late developmental stages of third molars [10]. Inter- and intra-observer reliability was tested, intra-observer reliability by re-examining 37 dental panoramic radiographs after 2 months. The agreements were quantified using Kappa and weighted Kappa statistics.

Three approaches were applied: using only the PT, using only the TM and using both PT and TM. The analyses were performed on a dataset with all subjects having at least four of the seven permanent index teeth and at least one third molar. For all three approaches, PT, TM and PT + TM, a Bayesian age-estimation model for the multivariate distribution of the stages conditional on age (multivariate continuation ratio model) was fitted as described in Boldsen et al. [56] and Fieuws et al. [57]. The probability density function using Bayes theorem was applied where the posterior distribution of age presents the prediction intervals which were presented as estimated likelihood curves. Point estimates for age were the maximum likelihood estimates. In the forensic age estimation, prior distribution could be constructed from previous information of the ages of dental developmental stages. In particular, transition analysis with ordinal data referring to an estimation procedure, where the direction of sequence of development is fixed and inferences about one stage to the next can be made, was used. The continuation ratio model for ordinal data models the probability of one particular category given the categories preceding it. The benefit of the method is that the approach is applicable for multiple age indicators. Therefore, the product of univariate likelihoods is used, and the resulting too small prediction intervals were corrected by the ad hoc procedure described by Boldsen et al. [56]. Three models were established for males and females separately and validated using 10-fold cross-validation. The sample was partitioned in ten subsets. The analysis was performed on one subset (training subset) and validated on the remaining subset (validation subset). This procedure was repeated on each subset and related remaining subset, and the obtained results (*n* = 10) were averaged [58]. The age-estimation performances were quantified by calculating the mean error (ME; true age minus predicted age), the mean absolute error (MAE) and root mean squared error (RMSE). The coverage of the obtained 95% prediction intervals was tested. Results were calculated over the total age range and in 1-year age intervals.

The described approach was implemented with SAS software (version 9.4 of the SAS system for Windows, SAS Institute Inc., Cary, NC, USA) using PROC NLMIXED to fit the continuation-ratio models and to evaluate the conditional likelihoods.

## Results

The intra- and inter-observer Kappa and weighted Kappa values revealed an excellent level of agreement for both PT and TM staging (Table 2).

**Table 2:** Intra- and inter-observer agreement using Kappa and weighted Kappa values and their 95% confidence intervals

Kappa statistics								
	Intra-observer				Inter-observer			
	PT		TM		PT		TM	
	Value	95% CI	Value	95% CI	Value	95% CI	Value	95% CI
Simple Kappa	0.95	0.92; 0.98	0.94	0.89; 0.99	0.97	0.95; 1.00	0.94	0.89; 0.99
Weighted Kappa	0.98	0.96; 0.99	0.97	0.95; 0.99	0.99	0.98; 1.00	0.97	0.95; 0.99

*PT* permanent teeth 31–37, *TM* third molars, *95% CI* 95% confidence interval

The overall age prediction performance results are presented in Table 3. On average, the combined approach (PT + TM) performed best, overestimating age by only 0.031 years (11.3 days) in males and 0.011 years (4.0 days) in females. Between approaches, all differences in ME, MAE and RMSE were significant (*p* < 0.0001 for ME and *p* < 0.05 for MAE and RMSE).

**Table 3:** Average sex-specific age-prediction performances of linear continuation ratio models using permanent teeth, third molars, and the permanent teeth and third molars combined

		Sex	
		Male	Female
Model	Value	*N* = 397	*N* = 406
PT	ME	− 0.260	− 0.291
	MAE	1.025	1.110
	RMSE	1.391	1.499
TM	ME	0.290	0.298
	MAE	1.390	1.301
	RMSE	1.787	1.735
PT + TM	ME	− 0.031	− 0.011
	MAE	0.852	0.909
	RMSE	1.095	1.175

All reported values are expressed in years

*N* number of subjects, *PT* Permanent teeth 31–37, *TM* Third molars, *ME* mean error, *MAE* mean absolute error, *RMSE* root mean square error

**Fig. 1:**
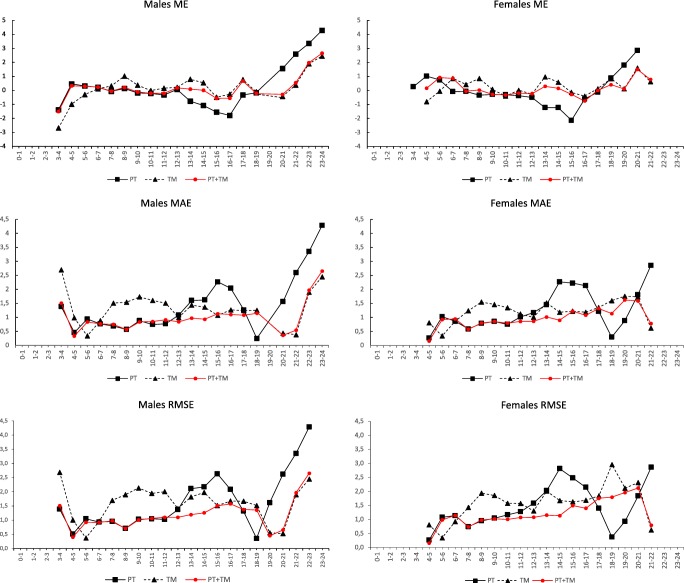
Sex-specific dependence of mean error (ME) (true age minus predicted age), mean absolute error (MAE), and root mean squared error (RMSE) on age. All reported values are expressed in years. PT Permanent teeth 31–37, TM Third molars, PT + TM permanent teeth and third molars combined

Considering the performances per age category of 1 year, with the approach combining PT + TM, the ME was small and constant in the age groups from 4 to 15 years in males (− 0.222 to 0.332 years) and from 7 to 16 years in females (− 0.327 to 0.294 years). In the PT approach, a large negative ME was observed in the age range from 15 to 17 years in males (− 1.791 to − 1.557 years) and in the 16-year age category in females (− 2.133 years), as well as a sudden drop in MAE and RMSE in the 18-year age category in both sexes. In the TM approach, the ME values had moderate variation in the age categories from 9 to 21 years in males (− 0.486 to 0.794 years), whereas in females, more variation was present (− 0.406 to 1.606 years) in respective age categories (Fig. 1).

For TM and PT + TM, the overall coverage of the 95% prediction interval fell within 1% of the interval in males and females (males 94.21% for TM and 94.96% for PT + TM; females 95.32% for TM and 94.58% for PT + TM). For PT, the overall coverage of the 95% prediction interval was 92.95% in males and 92.86% in females.

**Fig. 2:**
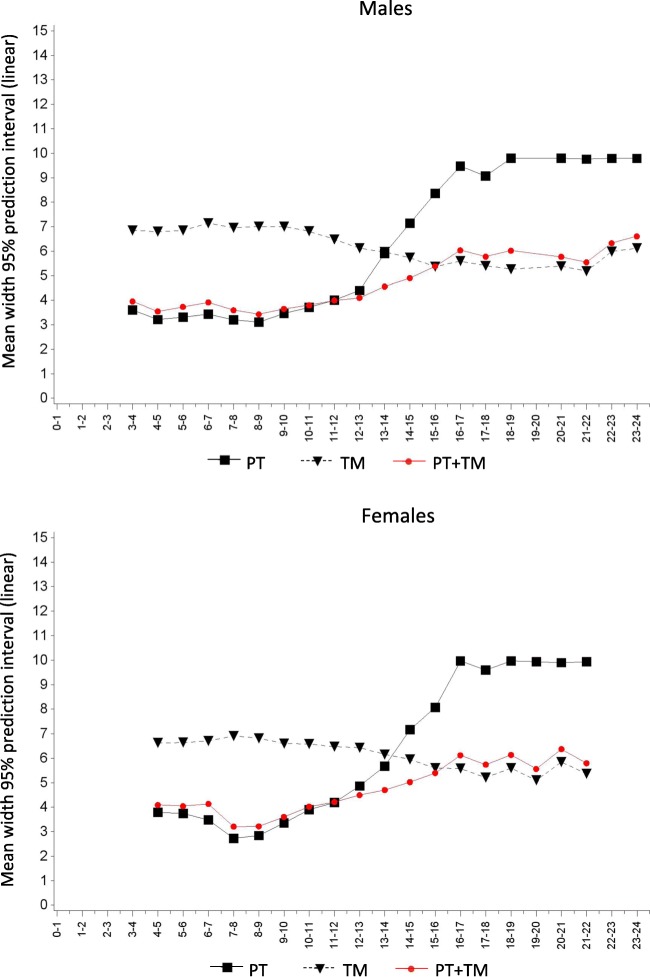
Mean width in years of the 95% prediction interval in males and females in permanent teeth, third molars, and permanent teeth, and third molars combined

The width in years of the 95% prediction intervals was most optimal in the 1-year age categories from 12 to 16 years for PT + TM in both males and females. Furthermore, the width in years of the 95% prediction intervals was larger in the 1-year age categories up to the age of 14 years for TM and was largest in the 1-year age categories above 14 years for PT (Fig. 2).

## Discussion

The first study hypothesis was that in a Somali sample the age prediction performances will improve by combining data of permanent teeth (PT) and third molars (TM) development. The hypothesis was not rejected —the PT + TM approach compared to PM and TM alone performed the best, overestimating age by only a mean of 11.3 days in males (equal to an ME of − 0.031 years) and a mean of 4.0 days in females (equal to an ME of − 0.011 years). This approach also yielded the lowest magnitude and variability of error (MAE and RMSE; Table 3). The ME provides information about the direction of the error, whether the model over- or underestimates the age of an individual. The MAE quantifies the magnitude of the error and RMSE the variability in error, assigning the most weight to the largest errors. In this sample, use of only third molars for age estimation led to the biggest error and variability.

When only using PT, the sudden drops in MAE and RMSE in the age categories 18 and 19 years are explained by the point estimate for subjects with fully developed permanent teeth, which equals 18.74 years for males and 18.63 years for females but is also due to a quite small number of individuals older than 17 years in the present Somali sample. The large negative error in the age range of 15 to 17 years in males and 16 to 17 years in females is due to the majority of subjects in this category having all their permanent teeth fully developed, third molars excluded, and hence receiving a maximum age prediction.

The second study hypothesis was that dental age predictions in a Somali population behave similarly to comparable age predictions in other studied populations. An abundant number of age estimation outcomes in Black populations have been reported and discussed in the literature [12, 20, 33, 35, 40, 41, 43–46, 48–50, 59–63], but they are not comparable with the present study due to differences in study set-ups, sampling, variation in age distribution, teeth considered, age predictors used and staging techniques and age estimation methods applied. Models combining PT + TM have been validated in United Arab Emirati, Brazilian, Japanese and Malaysian children and sub-adults [27–30]. In these studies, the RMSE was reported because it quantifies the variance in errors, assigning large errors more weight. In Brazilian children, the PT + TM approach resulted in RMSE values very similar to PT alone. Slightly more accurate age prediction when using the combined approach was achieved in females aged 14–15.99 years with a decrease in RMSE of 0.22 years [28]. Additionally, in Malaysian children, in the age categories between 14 and 16 years, the difference in RMSE decreased by 0.60 years in males and 0.34 years in females in the combined model (PT + TM) when compared to the PT model [30]. A similar trend was detected in the present study. The RMSE values were smaller in the PT + TM approach than in PT alone in the age categories 12–16 years in males and 10–16 years in females (Fig. 1). The reported RMSE using the four third molars (TM) alone has varied from 1.60 in females to 2.16 in males in studied United Arab Emirati [27], Japanese [29] and Malaysian [30] children and sub-adults, very similar to the present study with RMSEs of 1.735 in females and 1.787 in males. In the compared groups, TM provided the least accurate age prediction performances. Comparison of the present data with previous studies from other populations indicates that the second hypothesis was also correct; the three different age prediction approaches studied here perform equally in different ethnic groups. Thus far, the Malaysian children, in whom the average RMSE values were higher when using the combined model than the model for permanent teeth only comprise the only exception [30]. In general, the combined approach has indicated its applicability regardless of place of birth or place of residence.

The performance of PT + TM combined is optimal between 13 to 14 years of age in males and 12 to 14 years in females (Fig. 1), most likely because both PT and TM are developing at these ages. According to the literature, the age at completed permanent teeth development (up to the second molars) is, on average, 16 years [19, 64]. The present Somali data is in agreement with that of the 16-year-olds, 80.7% of males and 96.4% of females had all mature PT. Since all PT can be mature at the earliest around 12–13 years (here, the minimum in males was 13.39 years and in females 12.89 years), the use of the PT + TM approach beginning from the age of 12 years is likely to be beneficial compared to the PT approach. Indeed, the combined PT + TM approach indicated its superiority here regarding the 95% coverage results in the age categories from 12 to 16 years in both sexes (Fig. 2). Consequently, in forensic age estimation practice, the most benefit of combining PT and TM information will be obtained in 12- to 15-year-olds. In practice, development of a reference database and software for the utilisation of both permanent teeth and third molars data for increased accuracy of age estimation in this age group would be valuable. A method applying combined information from a large database of third molars and skeletal development, BioAlder, is already in use in Norway [65]. The oldest ages for immature PT were 17.37 years in males and 17.97 in females. After the age of 17 years in males and 19 years in females, the TM model performs equally well to the combined model (Fig. 1).

A previous study of the same Somali sample [44], comparing Willems model using Belgian Caucasian population [58] and the constructed Somali model, provided support for the universal application of the Willems model for forensic age estimation, since only small differences in age prediction performances were detected between the two models.

Limitations of the study were the relatively small size and uneven age distribution of the sample. Since the dental panoramic radiographs were retrospectively selected from collections taken for dental care, a limited number of radiographs were available in the youngest age groups. Individuals older than 17 years were also few, because only Somalis born in Finland were eligible. The subjective assessment of the crown or root lengths in the Köhler staging [21] might be problematic for some stages. For instance, in *crown half mineralised* (Cr1/2) and *crown 3/4 mineralised* (Cr3/4), individual variation in crown height and root length must be taken into account. However, in the present study, this was not reflected in the performed reliability tests. Since the study was performed in Finland with living conditions different from Somalia, socio-economic factors like nutrition may have affected the outcome. Previous studies have reported advanced dental development in overweight or obese children [66, 67], whereas Elamin and Liversidge [68] found no significant difference in dental development between malnourished and normal BMI groups of young Arabs in Sudan. The main strength of our study was its setting, offering the possibility to gather reliable data on individuals of ascertained ethnic background, general health and chronological age, which may not necessarily apply to studies performed locally.

In conclusion, the age prediction performance in Somali children, born and living in Finland, improves by combining the information of PT and TM, especially in the age groups of 12 to 15 years, when both PT and TM are still developing.
